# European starlings use their acute vision to check on feline predators but not on conspecifics

**DOI:** 10.1371/journal.pone.0188857

**Published:** 2018-01-25

**Authors:** Shannon R. Butler, Esteban Fernández-Juricic

**Affiliations:** Purdue University, Department of Biological Sciences, West Lafayette, Indiana, United States of America; Tokai University, JAPAN

## Abstract

Head movements allow birds with laterally placed eyes to move their centers of acute vision around and align them with objects of interest. Consequently, head movements have been used as indicator of fixation behavior (where gaze is maintained). However, studies on head movement behavior have not elucidated the degree to which birds use high-acuity or low-acuity vision. We studied how European starlings (*Sturnus vulgaris*) used high-acuity vision in the early stages of visual exploration of a stuffed cat (common terrestrial predator), a taxidermy Cooper’s hawk (common aerial predator), and a stuffed study skin of a conspecific. We found that starlings tended to use their high acuity vision when looking at predators, particularly, the cat was above chance levels. However, when they viewed a conspecific, they used high acuity vision as expected by chance. We did not observe a preference for the left or right center of acute vision. Our findings suggest that starlings exposed to a predator (particularly cats) may employ selective attention by using high-acuity vision to obtain quickly detailed information useful for a potential escape, but exposed to a social context may use divided attention by allocating similar levels high- and low-quality vision to monitor both conspecifics and the rest of the environment.

## Introduction

Animals generally gather visual information through visual fixations, whereby they maintain gaze on a single spatial location. Fixation is often accomplished by aligning the centers of acute vision (e.g., fovea; [1,2]) with a stimulus of interest for a certain period of time. Fixating with the center of acute vision allows the animal to obtain high visual resolution information [3]. However, the downside is that the center of acute vision generally occupies a small region of the visual field [4], and consequently animals are forced to move their eyes, heads, or bodies around to get snapshots of higher visual acuity from different parts of their visual surroundings [5,6]. An alternative is to use regions of the retina that are not the center of acute vision (non-foveal regions of the retina), which are expected to gather lower quality visual information. This could allow for quick detection of stimuli in a large proportion of the visual field, but at the expense of information with lower visual resolution [4]. Even after visual detection with retinal areas of lower visual acuity, animals tend to move their centers of acute vision towards the spot where the stimulus was detected in the first place (orienting response, [7]). The implication is that fixation is a fundamental process by which animals collect key information for decision making.

In birds, and particularly those with laterally placed eyes, the center of acute vision is moved around through rapid eye and head movements (reviewed in [4,6]). In general, for birds, head movements usually have a larger amplitude and occur much more frequently compared to eye movements [8–10] making the head a good proxy for gaze direction. One reason for this is that species with laterally placed eyes have their centers of acute vision projecting fronto-laterally (projections on either side of the head; [11]) compared to species with frontally placed eyes (parallel projections to the front of the head). Consequently, animals with laterally placed eyes must move the head to a larger degree to gather information on a given object with the right and left centers of acute vision (since they project to two different points in space). Consequently, head movements have often been used as proxies of fixation behavior in birds [12–14].

Head movement behavior has been studied in multiple contexts. Birds increase their head movement rates when exposed to a predator compared to a non-predatory heterospecific [15] and when the perception of risk is higher, such as when a bird is at the edge rather than the center of a flock; [16]. Other studies found that birds increase their head movement behavior after detecting food sources [17] and when using tools to obtain food items [18]. Additionally, in social scenarios, birds move their heads to collect information on the presence [19] and even gaze direction [20] of group mates. The general interpretation is that when birds are about to interact with an object, they move their heads laterally, realigning their centers of acute vision relative to the position of the object, and also relative to the position of the body. However, these studies have not provided much insight as to the degree to which birds use high-acuity vision while interacting with an object. One of the main limitations has been the need to understand where the centers of acute vision project, which can vary substantially between species [11].

To address this gap, we investigated the use of high-acuity vision in anti-predator and social contexts. We chose the European starlings (*Sturnus vulgaris*) as our model species because they (a) have laterally placed eyes [21], (b) have a single center of acute vision (fovea) per retina projecting fronto-laterally [22], and (c) are known to pay visual attention to conspecifics [20] and predators [23]. In general, we predicted that starlings would use their high-acuity foveal vision more than expected by chance to look at objects of interest in the early stages of visual exploration because they would be able to extract more high resolution information per unit time that can aid in quick decision-making (e.g., approach, move away). Additionally, we tested whether individuals would show laterality while moving the head, as it has previously been demonstrated that starlings may favor their left eyes in learning and visual discrimination tasks [24,25]. We predicted that starlings would prefer the left eye over the right eye because the former has a higher density of single cones, which are involved in chromatic visual resolution of non-moving stimuli [26].

## Methods

### Capture, husbandry, and care

We captured the birds in December 2013, using modified walk-in decoy traps at a landfill in Hamilton County, Ohio (N 39.2833, W 84.5947) with the assistance of the United States Department of Agriculture, Animal and Plant Safety Inspection Service (USDA, APHIS). There is no permit required in the state of Ohio to catch European starlings, or to bring them to Indiana because the capture of this invasive species is not regulated by the federal government. We kept birds in mixed-sex groups in aviaries (2.5 x 2.5 x 3.5 m) outdoors at the Ross Biological Reserve (Tippecanoe Country, IN, N 40.4167, W 87.0693). The Ross Biological Reserve is owned and maintained by the Purdue Department of Biological Sciences and we had permission to house our birds. We provided care daily and fed the animals ad libitum: cat food, game bird maintenance chow and water. Birds had access to heated water available in the winter months. We ran this experiment between September 2014 and February 2015. Although birds were primarily housed outdoors, a few days before trials we moved them to indoor enclosures in groups of 2–4. To reduce stress, each bird participated in trials every other day, at most. Animals that participated in this study were also part of another experiment where we assessed their scanning behavior when perching in pairs [19], but we do not believe that this experiment affected their behavior in the current study. All animal capture, care, and use procedures were approved by the Purdue Institutional Animal Care and Use Committee (IACUC protocol 1306000876). No animal became sick, injured, or was euthanized during this study. At the end of the study, animals were transferred to other studies or purposes following IACUC recommendations as European starlings are considered an invasive species and cannot be released in the wild the US.

### Experimental procedures

Probably, the most precise way of measuring fixation is by using an eye tracking device which tracks eye movements and the projection of the center of acute vision onto the visual surroundings of an animal. Eye tracking technology has been successfully used in primates [27], rats [28], dogs [29], etc. Although eye tracking technology has been developed in birds [23,30], it is not yet to the point that we could utilize it to track the movements of both eyes simultaneously in small birds that can move freely. The available avian eye trackers only work in larger species than the starlings, and they can only track one eye at a time [10,30]. Other eye tracking technology for species with a similar size to that of starlings require restraining body and head movements [23]. Given these technological limitations, we chose to use head movements as a proxy of fixation behavior. Head movements are actually a commonly used proxy of fixation and gaze shifts in birds [5,10,19,31–33].

**Fig 1:**
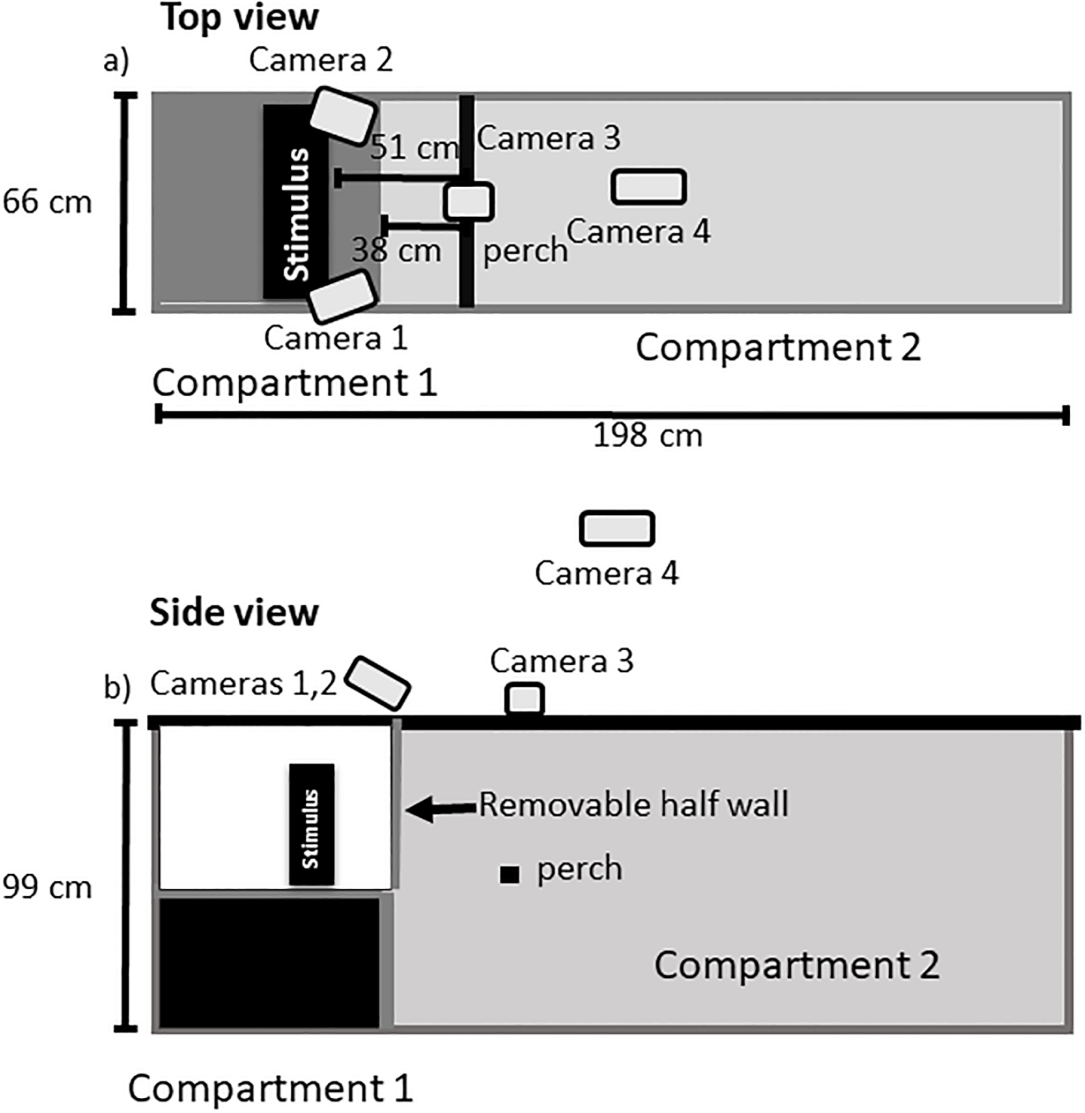
Experimental arena set up from (a) top and (b) side views.

Our experimental approach basically consisted of exposing a single subject to a given visual stimulus present (cat, hawk, conspecific) or absent in an experimental arena with a patch of artificial grass. The arena was 198 cm long x 66 cm wide x 99 cm high, and divided in two compartments by a partition that was placed perpendicularly to the longer edge of the arena (Fig 1a and 1b). The partition was removable. Compartment 1 was 38 cm long (and 66 cm wide x 99 cm high) and contained the visual stimulus. Compartment 2 was 166 cm long (and 66 cm wide x 99 cm high) and contained a perch that was 50 cm above the bottom of the arena and 38 cm back from the removable partition (Fig 1a and 1b) for the bird to perch on. The arena was monitored by four cameras (Fig 1a and 1b). One camera was directly over the birds head, two cameras were positioned to monitor the front of the bird, and a fourth camera was above the entire arena for a top-view.

We used 30 European starlings (16 males, 14 females). Each individual was exposed to four different treatments: a cat on a patch of grass, a taxidermy hawk (Cooper’s hawk, *Accipiter cooperii*) in a perching position on a patch of grass, a stuffed European starling on a patch of grass, and an empty patch of grass. To minimize simple pseudoreplication of the visual stimuli [34], we had 2 exemplars of a cat and 4 exemplars of a starling. Unfortunately, we had available one only exemplar of a perching hawk due to the difficulty in obtaining taxidermy raptor specimens. All of our stimuli were 3 dimensional objects (pictures are available in the supplemental information S1a–S1e Fig). The cats were ordered from Piutre`Animal Collection (S1a Fig) and Hansa Toy Store (S1b Fig) and were made of synthetic polyester fur and plush material (see also [11, 35]). Our taxidermy hawk was borrowed with permission from the Purdue Department of Forestry and Natural Resources. Our model conspecifics were made of skins from European starlings that had previously died due to health reasons and were frozen to keep them intact (see [20] for methods). Previous studies have shown that birds perceive models as live animals [36,37]. Specifically, starlings [37] perceive stuffed model of the same species as conspecifics and taxidermy mounts of raptors have been shown to elicit their antipredator behaviors [38–41]. Consequently, we believe we used a valid experimental manipulation.

Furthermore, we chose a repeated measures design so that the statistical models could parse out the variation between stimuli from the variation that naturally exists between individuals. One concern with repeatedly exposing the same individual to the same stimulus over time is habituation, but we tested for this effect. When starlings do not habituate to a stimulus, they tend to engage in escape behaviors, such as flying [42]. We used whether an individual engaged in flying behavior within the experimental arena as a proxy of habituation to the stimuli and asked whether trial order was a significant predictor of flying behavior, after controlling for the identity of the stimulus and the interaction between trial order and the identity of the stimulus. If the birds habituated, we would expect that the probability of flying behavior would significantly decrease over trial order as birds habituated. This effect could show up either in the main effect of trial order (i.e, if birds habituated to exposure to stimuli in general) or the interaction between stimulus and trial order (i.e. If birds habituated to some stimuli and not others), so we tested both of these possibilities.

A trial began when we released a bird into compartment 2. Then, we hid out of the bird’s sight and watched the bird on a computer screen. The computer screen was connected to a DVR (Night Owl, H264 4-channel DVR), which was connected to 4 cameras, as described above (Fig 1a and 1b). The purpose of the DVR was to synchronize the 4 videos feeds so that we knew exactly when the stimulus was visible to the animal. The observer waited until the bird was perched with both feet oriented forward (towards the removable partition) for 5 consecutive s, which was a requirement for the treatment to be presented, to ensure that the animal settled down. If the bird did not perch while oriented forward for 5 consecutive s during a period of 15 mins (requirement not met), we decided to terminate the trial and attempt it on a different day. After the 5 s perching period (requirement met), we removed the partition by slowly sliding it down and exposed the subject to the treatment. We recorded its behavior for 30 s and afterwards finished the trial, which lasted from 1 to 16 mins depending on how it took for the bird to meet the 5 s perching requirement to become settled.

During the 30 s after the treatment exposure, the subject would be perching, on the ground or flying around. Therefore, for behavioral recordings, we only used the first 5 s (since the partition was removed) of cumulative perching time to make sure the animal was in a position where it was perching with its beak towards the stimulus since then it could use either high- or low-acuity vision. In some cases, the subject would spend these 5 s perching and visually exploring the stimulus without interruptions. In other cases, the subject would go back and forth. For instance, the subject would spend 3.5 s perching after the partition was removed, but then suddenly leave, and we would pause recording until it would come back and resume recording for another 1.5 s until we accumulated 5 s of recording time.

We coded the videos using two programs: Tracker (http://physlets.org/tracker/) and virtualdub (http://virtualdub.org/). Using virtualdub, we measured all head movements that occurred within the first 5 s of perching time after treatment exposure. We excluded head movements that were associated with landing and taking off. Using virtualdub, we recorded each frame when the subject’s head became still (did not change position for 2 or more consecutive frames or 67 ms, at 30 frames per s) indicating a fixation after a head movement. We chose this time frame because it was the highest resolution that we could measure based on our cameras (2 frames per s at 30 frames per s yields 67 ms). However, we believe that this resolution of our analyses was more than sufficient because it was finer than the average eye fixation duration in starlings (700–900 ms, [23]), ensuring that we recorded all of the fixations that the subject made. Then, an undergraduate student measured the angle of the head at each previously marked frame using Tracker. After self-training and under the supervision of SRB, this student was consistent with herself within 2° for 95% of her measurements. Inter-observer bias was not a problem because this student measured all the head angles for this study, as well as previous studies we conducted. Finally, SRB measured the position of the bird on the perch, using virtualdub. Due to the need to synchronize our cameras with each other into a single video stream, it was not possible for us to code the trials blindly.

Using the angle of the head, the position of the bird on the perch relative to the stimulus, the configuration of the starling visual field, including its eye movement amplitude [21], and the location of its center of acute vision in the retina [22], we projected the fovea onto the plane in front of the bird where the stimulus was located, using the inverse tangent function (details in [43]) to determine whether the bird was aligning its fovea on the stimulus or not. We repeated this analysis each time the bird kept its head still (i.e., fixated, see criterion above) during the sampling period. We then calculated the proportion of fixations where the fovea was on the stimulus (or the region where the stimulus would be for the stimulus absent treatment) for each trail, and also marked whether it was with the left or the right fovea (high acuity vision; area of the visual field where the center of acute vision projected into the visual field), or with the non-foveal region (low acuity vision; any other area of the visual field that the fovea did not project into). We determined the chance levels of the fovea being on the stimulus by measuring how often the fovea was on the region that the stimulus (cat, hawk, conspecific) would be in the stimulus absent treatment (following [30,36]). We chose this approach, as an alternative to assuming a uniform or random a priori distribution of fixation points, because it accounts for differences in the size of our stimuli as well as any potential bias of the bird looking into a particular region of the enclosure. For example, the cat is much larger than the conspecific, so by random chance alone, the fovea would be more likely to land on the cat than the conspecific as it takes up a larger area of the visual field. Our approach makes the potential area of fixation points in the stimulus absent treatment the same as the stimulus present treatment. In our set up, it was physically impossible for the bird to get its left and right fovea on the stimulus at the same time due to the width of the arena and the distance that the bird was from the stimulus. Therefore, each foveal fixation could be classified as either left fovea, right fovea, or non-foveal.

### Statistical analysis

We used general linear mixed models to analyze our data. First, we asked whether overall head movement rate differed between stimuli since head movement rate (number of head movements made/s) has been used extensively in the literature on avian head movement behavior (e.g., [15,32]). For this analysis, head movement rate was the dependent variable and treatment (cat, hawk, conspecific, empty) was the independent variable. We controlled for sex since it has been shown to affect head movement rate in some birds [32].

We also ran one model for each of the three visual stimuli (cat, hawk, conspecific), with the dependent variable being the proportion of fixations with the fovea directed onto the portion of the experimental arena where the stimulus was positioned in the stimulus present treatment, as well as the same area it would have occupied by chance in the stimulus absent treatment. The independent variables were whether or not the stimulus was present, and sex of the bird (male, female). We ran three additional models (again, one for each stimulus) with the data in which the stimulus was present to compare the proportion of fixations that occurred with the left vs the right fovea. For these models, the dependent variable was the proportion of fixations directed towards the stimulus with either fovea, and the independent variables were eye (left, right), and sex (male, female). We randomized the order of the treatments for each bird. We were not able to include trail order in our model because using an autoregressive variance-covariance matrix in our repeated measures statement resulted in not having enough degrees of freedom for the model to converge. Additionally, we tested for habituation effects using a generalized linear mixed model with whether or not an individual engaged in flying behavior as the dependent variable (0/1), and stimulus, trial order, and the interaction between stimulus and trial order being the independent variables. All analyses were repeated on individual to avoid pseudoreplication and run in SAS v. 9.4 assuming equal variance between measurements. We set our threshold of significance at 0.05 (alpha-level), meaning that if the p-value was below this level, we rejected our null hypothesis of no significant difference.

## Results

We found that overall head movement rate did not differ significantly between treatments (F_3,59_ = 0.4, P = 0.755; means ± SE in head movements per s: cat: 2.5 ± 0.71, hawk: 1.4 ± 1.2, conspecific: 1.5 ± 1.3, stimulus absent: 1.2 ± 1.3), nor was head movement rate affected by sex (F_1,28_ = 1.14, P = 0.295).

**Fig 2:**
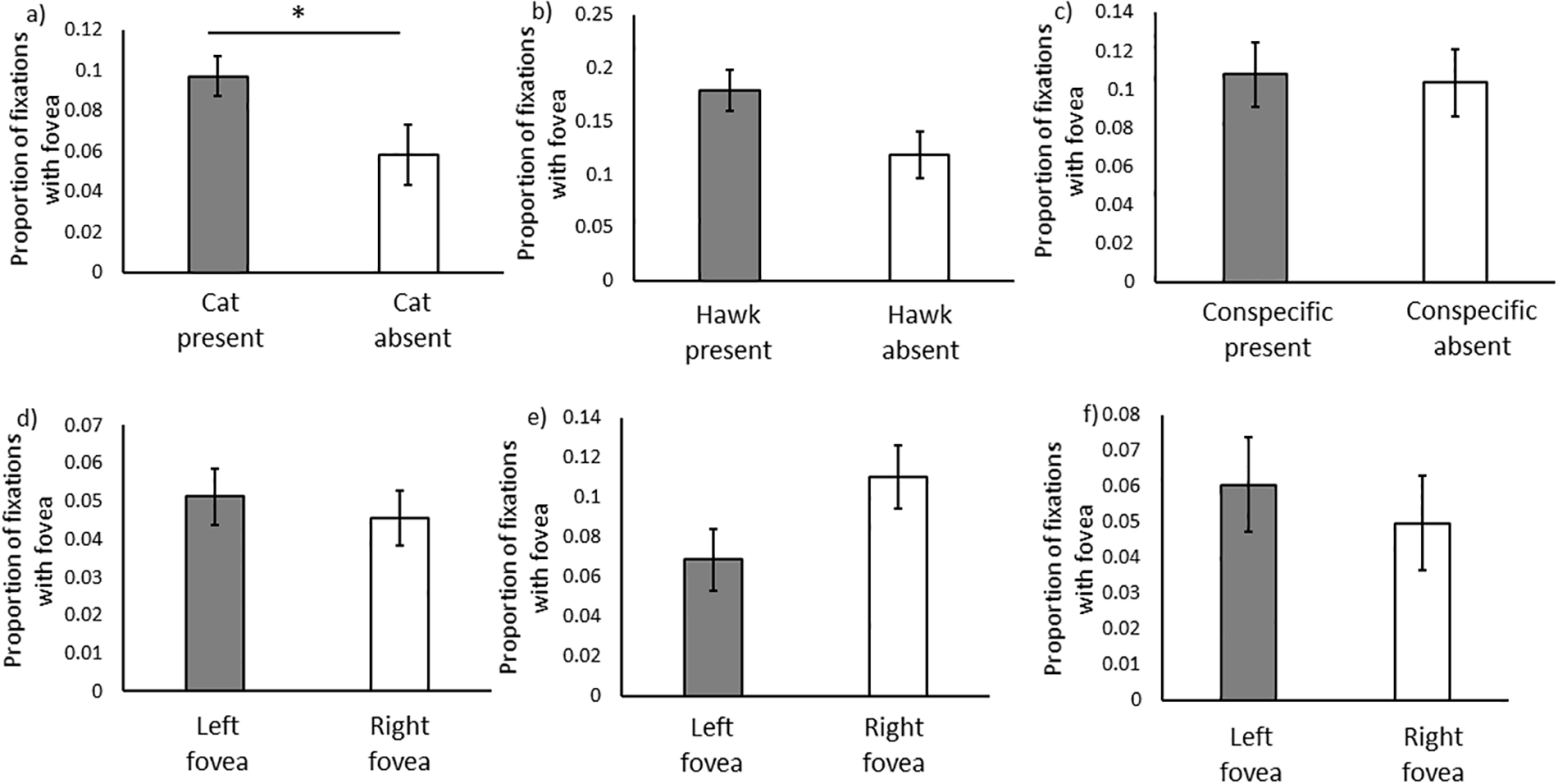
The proportion of fixations made with the center of acute vision (right and left foveae) when (a) the cat was present or absent, (b) the hawk was present or absent, (c) the conspecific was present or absent. Additionally, we present the proportion of fixations made with the left or right center of acute vision when (d) the cat was present, (e) the hawk was present, and (f) the conspecific was present.

Starlings oriented their foveal vision towards the cat significantly more compared to chance levels in the same location when the stimulus was absent (F_1,17_ = 4.17, P = 0.045, Fig 2a). Although starlings tended to orient their foveal vision towards the hawk more than chance levels, this trend was not statistically significant (F_1,16_ = 4.42, P = 0.052, Fig 2b). However, starlings did not orient their foveal vision towards the conspecific more than chance levels (F_1,14_ = 0.03, P = 0.859 Fig 2c). There was no significant effect of sex on orienting the fovea towards any of the stimuli (cat, F_1,27_ = 1.73, P = 0.199; hawk, F_1,24_ = 1.20, P = 0.28; conspecific, F_1,23_ = 0.43, P = 0.520).

We did not find significant differences in the use of the left and the right foveal vision for any of the stimuli (cat, Fig 2d, F_1,27_ = 0.29, P = 0.592; hawk, Fig 2e, F_1,23_ = 3.62, P = 0.070; conspecific, Fig 2f, F_1,20_ = 0.34, P = 0.568) after controlling for sex, which was also non-significant for all stimuli (cat, F_1,26_ = 1.39, P = 0.250; hawk, F_1,24_ = 0.46, P = 0.504; conspecific, F_1,19_ = 1.07, P = 0.314).

We found that trial order did not affect whether starlings engaged in flying behavior (F_1,98_ = 0.05, P = 0.824), nor did it significantly interact with treatment to predict flying behavior (F_2,98_ = 2.28, P = 0.110). This suggests that starlings in our experiment did not habituate to the stimuli they were exposed to. This validates the fact that we did not include trial order in our other models.

## Discussion

We found that starlings tended to use their centers of acute vision more than chance levels to look at cats. Contrary to our prediction, however, starlings used their centers of acute vision at chance levels to look at conspecifics, suggesting they relied to a large degree on their low-acuity vision in this social scenario. Additionally, we did not find that starlings showed laterality in their use of the right or left center of acute vision. Despite previous work on head movement behavior in antipredator [15] and social [19] contexts, our study provides the first empirical evidence as to how birds with laterally placed eyes use their high-acuity vision to explore with both eyes different stimuli, even without variation in head movement rates. This was possible because our model species had its visual field [21] and position of the center of acute vision [22] characterized. Using species-specific visual configuration information is critical for interpreting these findings given the high degree of inter-specific variation in these traits (e.g., [12]).

Birds have been found to increase their head movement rates when exposed to a feline ground predator (e.g., chaffinches, [11]). Our results add a new dimension by showing that under a similar type of stimuli starlings use their high-acuity vision above chance levels to visually explore the cat model, likely to obtain high visual resolution information, which can be critical to anticipate the angle, speed, and trajectory of a potential attack. We considered a couple of factors that could have biased these results. First, the responses to the cat could be significant because it was the largest of the 3 stimuli, which could have increased the chances of the starling foveating where the stimulus was. However, because we made comparisons between each stimulus and because the control condition consisted of the area where each stimulus would have occupied if it had been there, we think the confounding effects of size were minimized. Second, the responses to the cat could be significant because the animals were prone to looking in the particular area of the enclosure where the stimulus happened to be. However, our control also excludes that possibility because by projecting where the stimulus would have been when there was not stimulus present, we were able to compare where the animal was looking with no such stimulus present. Overall, our control treatment and analytical procedures minimized both sources of potential bias.

When starlings were exposed to a perching Cooper’s hawk, they showed a similar trend in the use of high-acuity vision to that shown towards the cat, but it failed to be significant. Interestingly, chaffinches did not increase their head movement rate in response to a taxidermy sparrow hawk (aerial predator) either [15]. The lack of significance in our study could be due to multiple factors: (a) not enough statistical power to detect the signal, (b) lack of replication of the stimulus for the hawk as we were only able to borrow one exemplar of the hawk, and (c) an increase in eye movements (which we could not measure) rather than head movements. Alternatively, the way that starlings gather visual information in terms of investment in high-acuity vision may differ between different types of predators. However, we do not believe this may be the case because starlings have been shown to use their fovea to look at aerial predators using an eye tracker where subjects could move their eyes but not their heads [23].

Starlings did not use their high-acuity vision above chance levels to look at the conspecifics. In principle, this strategy may not seem advantageous from the perspective of gathering social information for foraging and anti-predator purposes, which starlings have been repeatedly shown to do [24,25,44,45]. However, in a recent study where pairs of starlings were perching side-by-side, individuals actually used their low-acuity vision to monitor its neighbor [19]. In the present study, starlings were perpendicular to each other in a “face-to-face” context, and they still did not actively engage in high-acuity vision to visually explore the conspecific. These two lines of evidence suggest that in a social context, starlings may benefit by keeping track of changes in the presence and behavior of neighbors through low-quality vision and releasing their high-acuity vision to search the visual environment for food or predators [19]. However, it is important to note that although starlings did not use high-acuity vision on the conspecific above chance levels, they still used it in multiple occasions (Fig 2c, non-zero values). These few instances may be enough for individuals to obtain fine detailed social information and make decisions about individual identity [46], foraging opportunities [47], kleptoparasitism [48], predator detection [37], etc.

Our findings have implications for selective attention (i.e., attending to some stimuli more than others) and divided attention (i.e., the difficulty animals have attending to multiple stimuli at once) [49,50]. First, by using certain regions of the retina for specialized tasks (e.g., detecting predators, monitoring conspecifics, foraging), animals could reserve other areas of their visual fields for the other tasks. Second, by limiting certain areas of the retina for use for one task and others for different tasks, animals could better divide up their attention between multiple locations without having to move their head and invest time and energy in stabilizing gaze. When exposed to a predator, such as a cat, starlings may be using selective attention by allocating the high-acuity vision to gathering detailed information and leaving the low-quality vision to monitor the rest of the environment. However, in social contexts, starlings may be using divided attention by using to similar degrees high- and low-quality vision to monitor both conspecifics and the rest of the environment. Future work should focus on testing these attention mechanisms in different ecological (e.g., variations in predation risk) and social (e.g., variations in group size, neighbor distance) conditions.

## Supporting information

S1 Fig Pictures of: a) stuffed cat example 1, b) stuffed cat example 2, c) stuffed conspecific, d) taxidermy Cooper’s hawk, e) close up of stuffed conspecific. (TIF)

S1 Video Video of starlings in presence of stuffed cat. (MP4)

S2 Video Video of starlings in presence of a stuffed conspecific. (MP4)

S3 Video Video of starlings in presence of a taxidermy hawk. (MP4)

S1 Dataset Data butler plosONE.xlsx. (XLSX)
