# Supplementary figures and images for: European starlings use their acute vision to check on feline predators but not on conspecifics
Source: PLoS One. 2018 Jan 25;13(1):e0188857. doi: 10.1371/journal.pone.0188857 (PMC5784912; doi:10.1371/journal.pone.0188857)

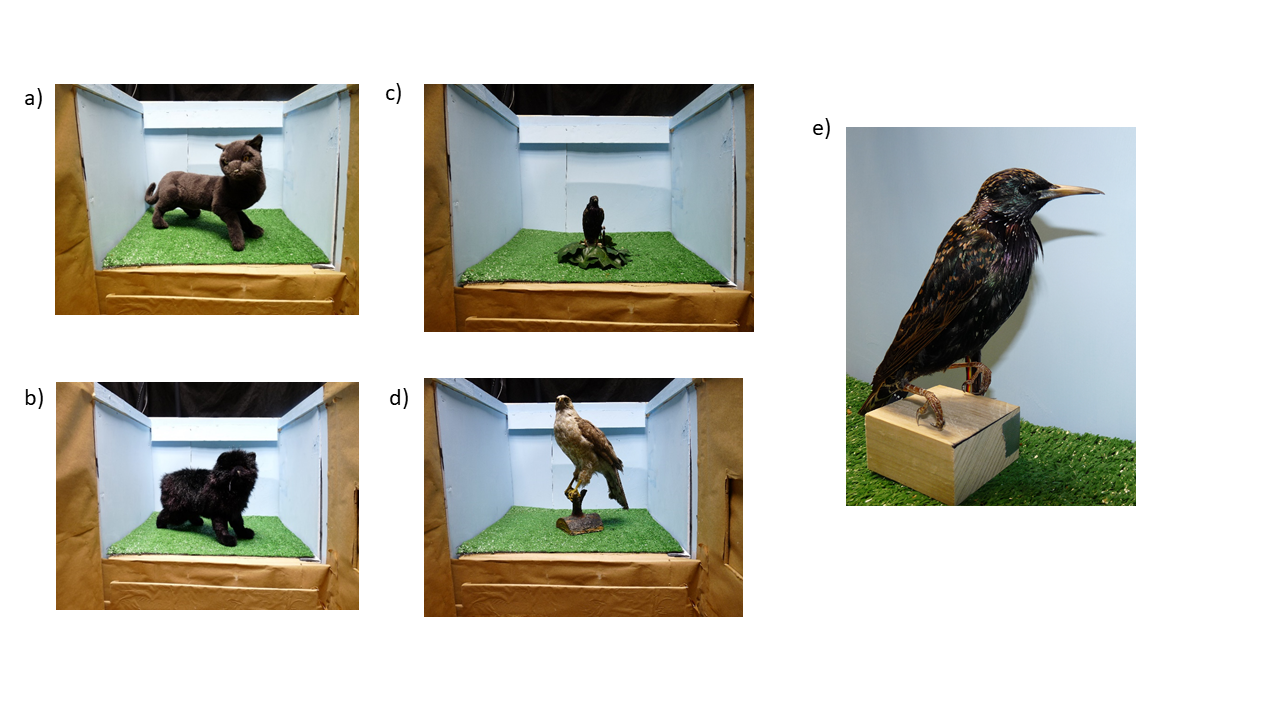

Supplement: S1 Fig — (TIF) [file pone.0188857.s001.tif]
